# Transcatheter Artery Embolization for Postoperative Haemorrhage after Arterio-Venous Malformation – Safer Option

**DOI:** 10.15388/Amed.2021.28.1.11

**Published:** 2021-03-09

**Authors:** Panda Subrat, Sharma Nalini, Khan Dina Aisha, Saha Anusmita, Das Rituparna, Phukan Pranjal

**Affiliations:** Department of Obstetrics and Gynaecology, North Eastern Indira Gandhi Regional Institute of Health and Medical Sciences Shillong, Meghalaya, India; Department of Obstetrics and Gynaecology, North Eastern Indira Gandhi Regional Institute of Health and Medical Sciences Shillong, Meghalaya, India; Department of Obstetrics and Gynaecology, North Eastern Indira Gandhi Regional Institute of Health and Medical Sciences Shillong, Meghalaya, India; Department of Obstetrics and Gynaecology, North Eastern Indira Gandhi Regional Institute of Health and Medical Sciences Shillong, Meghalaya, India; Department of Obstetrics and Gynaecology, North Eastern Indira Gandhi Regional Institute of Health and Medical Sciences Shillong, Meghalaya, India; Department of Imaging and Interventional Radiology, North Eastern Indira Gandhi Regional Institute of Health and Medical Sciences Shillong, Meghalaya, India

**Keywords:** Gestational trophoblastic Neoplasia, Haemorrhage, Complication, Hysterectomy, Arteriovenous malformation, Transcatheter artery embolization

## Abstract

**Summary. Introduction:**

Hemorrhage is one of the commonest and dreaded complications especially with pelvic surgeries. Gestational trophoblastic neoplasias (GTN) are notorious for their propensity to bleed torrentially and metastasis to vital organs. GTN is associated with an arterio-venous malformation (AVM) about 10-15% of the time, which can lead to bleeding after surgery or after complete remission. After the failure of conventional management with chemotherapy or surgery one is compelled to take another modality of management. One of such methods is the use of transcatheter artery embolization in cases of GTN or post-hysterectomy cases of GTN. Transcatheter artery****embolization (TAE) was effective in controlling bleeding due to arterio-venous malformation in 96% of cases.

**Case:**

46 years P2L2A5 (para 2, living issue 2, abortion 5) post-hysterectomy patient presented with bleeding from the vagina after surgery. Twice she underwent vaginal vault repair after hysterectomy but failed. Ultrasonography (USG) showed arterio-venous malformation (AVM); angiography revealed massive extravasation from (left internal iliac artery and abnormal vascularity from the right internal iliac. She was taken up for bilateral internal iliac arteries embolization but again had a heavy bout of bleeding after one week. CT scan confirmed a residual lesion and she underwent a repeat embolization after which the bleeding stopped. Serum BHCG was advised during workup and it was 1997 IU/ml. A diagnosis of GTN was confirmed. The patient was discharged after two cycles of chemotherapy with advice to review for the third one on an outpatient department basis.

**Conclusion:**

We concluded that TAE is an effective and safer alternative to surgery in postoperative bleeding from AV malformation in the case of GTN. It can be repeated and should be made to more liberal use in emergency settings.

## Abstract (lt)

**Santrauka. Įžanga:**

Hemoragija yra viena iš dažniausių ir grėsmingiausių komplikacijų, ypač dubens chirurgijoje. Gestacinės trofoblastinės neoplazijos (GTN) pasižymi stipriu kraujavimu ir metastazių išplitimu į gyvybiškai svarbius organus. Maždaug 10–15 proc. atvejų GTN yra susijusios su arteriovenine malformacija (AVM), dėl kurios galima nukraujuoti po operacijos arba po visiškos remisijos. Tuo atveju, kai tradicinis gydymas chemoterapija arba chirurginis gydymas nepavyksta, tenka imtis kitų gydymo metodų. Vienas iš tokių metodų yra transkateterinės arterinės embolizacijos naudojimas GTN atveju arba esant GTN po histerektomijos. Transkateterinė arterinė embolizacija (TAE) 96 proc. atvejų buvo efektyvi sustabdyti kraujavimą dėl arterioveninės malformacijos.

**Klinikinis atvejis:**

46 metų P2L2A5 (PARA2 – du gimdymai, du gyvi esantys palikuonys, penki abortai) pacientei po histerektomijos prasidėjo vaginalinis kraujavimas. Jai du kartus buvo atlikta makšties skliauto rekonstrukcija po histerektomijos, tačiau nesėkmingai. Ultrasonografija (USG) parodė arterioveninę malformaciją (AVM); angiografija atskleidė didelę ekstravazaciją iš kairės vidinės klubinės arterijos ir dešinės pusės vidinių klubinių kraujagyslių pakitimus. Pacientei buvo atlikta abipusė vidinių klubinių arterijų embolizacija, tačiau po savaitės vėl prasidėjo smarkus kraujavimas. Kompiuterinė tomografija patvirtino, kad yra likusi žaizdelė, ir pacientei buvo atlikta pakartotinė embolizacija, po jos kraujavimas liovėsi. Buvo rekomenduojama atlikti BHCG tyrimą patikros metu, o gautas rezultatas parodė 1997 IU/ml. Pasitvirtino GTN diagnozė. Pacientė buvo išleista į namus po dviejų chemoterapijos ciklų, rekomenduojama tikrintis ambulatoriškai dėl trečiojo ciklo.

**Išvada:**

TAE yra efektyvi ir saugesnė alternatyva chirurgijoje pooperaciniam kraujavimui dėl AV malformacijos GTN atveju. Ji gali būti pakartotinė ir turėtų būti liberaliau taikoma neatidėliotinais atvejais.

**Reikšminiai žodžiai**: gestacinė trofoblastinė neoplazija, hemoragija, komplikacija, histerektomija, arterioveninė malformacija, transkateterinė arterinė embolizacija

## Introduction

In all surgeries, there is the possibility of postoperative complications depending on the nature of the surgery. Hemorrhage is one of the commonest and dreaded complications especially with pelvic surgeries. Because severe hemorrhage is a major cause of postoperative morbidity and death, one has to deal with it promptly and meticulously. Many times one has to resort to the second surgery, which is an enormous challenge for the surgeon and anesthetist, especially under hemodynamically unstable conditions. Various surgical complications like infection, bleeding, and ureteric injury may occur in an emergency setting due to second surgery. Additionally, the pelvis is highly vascular with widespread anastomosis owning to which identifying and ligating these vessels is difficult and has a limited chance of successful hemostasis. Surgical evacuation of hematoma has the risk of “loss of tamponade” and further uncontrolled bleeding. Transcatheter arterial embolization (TAE) is an effective nonsurgical treatment for patients with obstetric and gynecologic hemorrhage. [1] However, there are very few reports on the efficacy of TAE in patients with postoperative hemorrhage after gynecologic surgery [2].

TAE was effective in controlling bleeding due to arterio-venous malformation in 96% of cases [3]. TAE is effective, avoids surgical risks, preserves fertility, has a lower complication rate and a shorter hospitalization. Saxena A et al. concluded that before embolization, angiography can greatly enhance the favorable outcome by determining the bleeding location [4].

Here we report a case of post-hysterectomy repeated hemorrhage due to arterio-venous malformation(AVM) in the case of gestational trophoblastic neoplasia (GTN) managed by TAE.

## Case Report

A 46 years old female, P2L2A5 (para 2, living issue 2, abortion 5), was referred to our hospital with complaints of multiple episodes of intermittent bleeding per vagina since she underwent a hysterectomy 2 and half months ago (8/8/2020). Indication of hysterectomy was heavy menstrual bleeding for two years. Before a hysterectomy, she underwent an endometrial biopsy. Histopathology report of endometrial biopsy, as well as hysterectomy specimen, was not available. Seven days following hysterectomy, she had episodes of heavy bleeding per vagina, and thus, she underwent repair of the vaginal vault twice. She continued to have vaginal bleeding on and off and was further evaluated by a contrast-enhanced MRI of pelvis, suggesting arterio-venous malformation (AVM) in the left pelvis near the vaginal vault. She was, thus, referred to our institute for further management.

**Figure 1.**
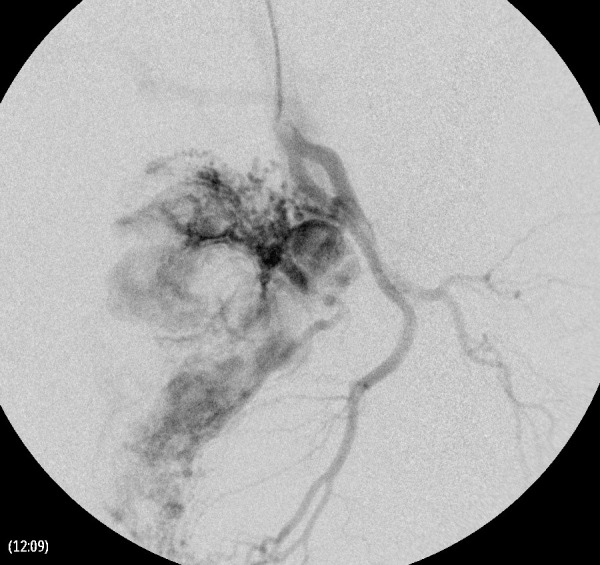
Angiography of left internal iliac artery demonstrates the abnormal vascular blush with arterio-venous shunting from the posterior division.

**Figure 2.**
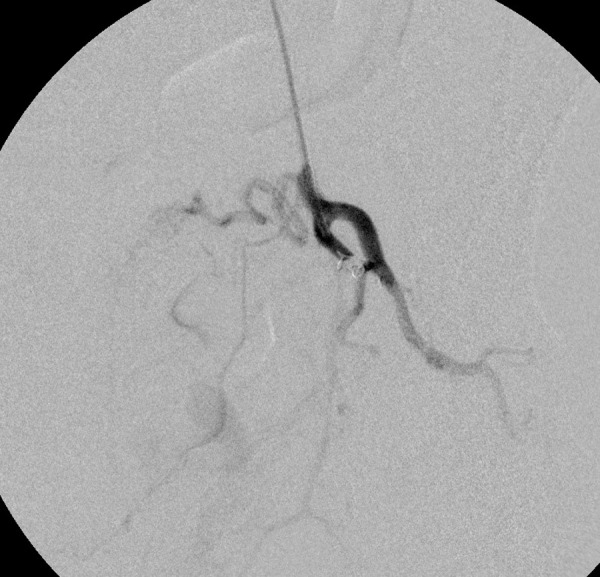
Immediately after angiography patient had severe bleeding per vagina. So a peripheral coil was deployed to reduce the bleeding.

**Figure 3.**
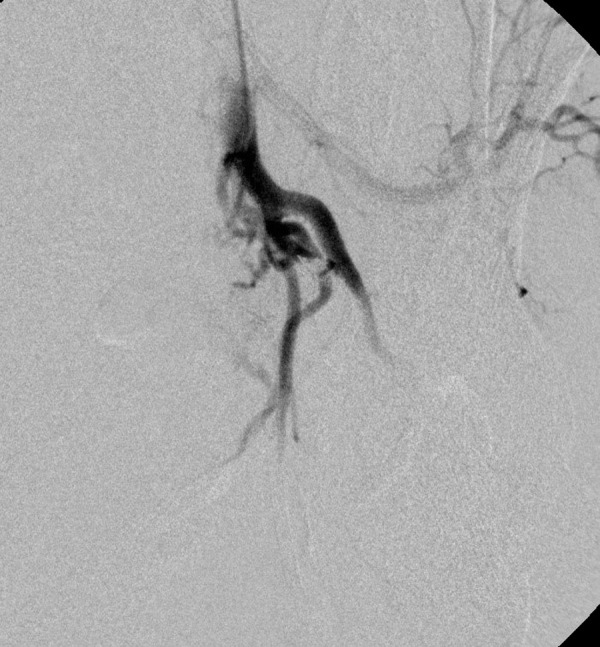
This was followed by embolization of the blush with 300 micron PVA particles.

**Figure 4.**
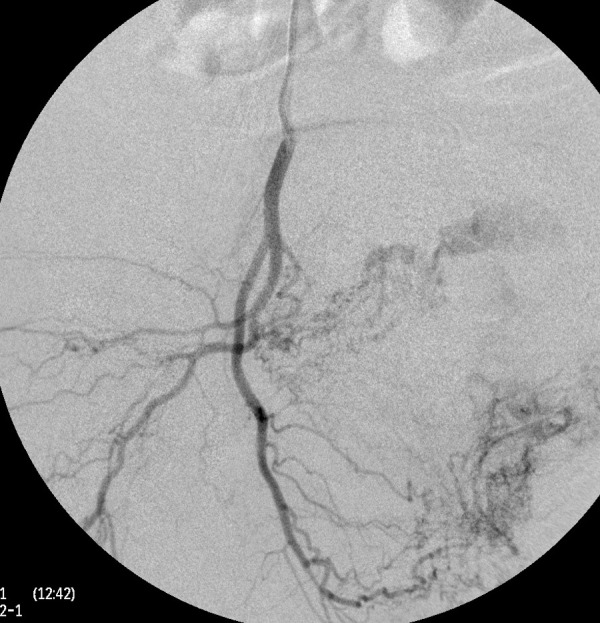
Angiography of the right internal iliac artery illustrates the abnormal vascular blush at the pelvic cavity with feeders from posterior division of the right internal iliac artery. The blush was embolized with 300 micron PVA particles.

**Figure 5.**
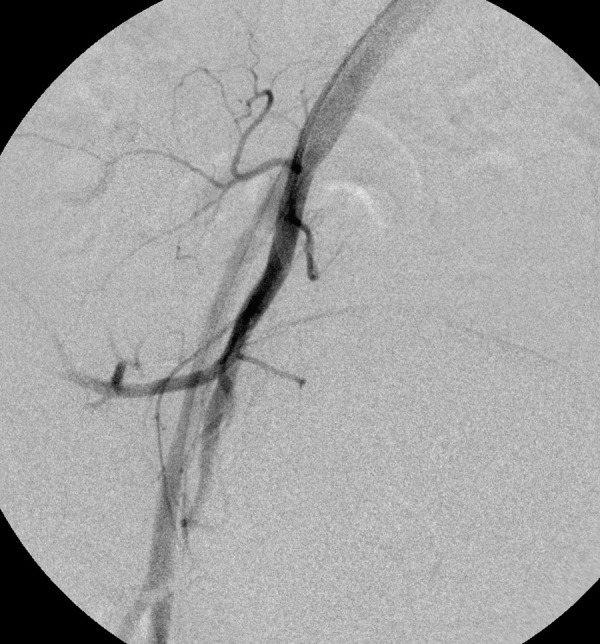
Post-embolization angiography reveals the complete obliteration of the blush

A thorough clinical history revealed that she had 5 induced abortions following last childbirth. She was also tested positive on a urine pregnancy test before hysterectomy. However, the patient could not reveal her exact diagnosis. On general physical examination, she had an average built with mild anemia. Her systemic examination findings were normal. On vaginal examination, the vault appeared unhealthy with no active bleeding. All routine investigations were sent including high vaginal swabs for culture and sensitivity, which were normal. Haemoglobin 11 gram/dl, PCV-33%, total leucocyte count 9.6×10^3^/cmm, platelet 200×10^3^/microliter, blood glucose 90 mg/dl, serum creatinine 0.8 mg/dl, SGOT-28U/L, SGPT-22U/L, TSH 2.8 miu/L, INR-1.1, ECG were normal. Ultrasonography with Doppler showed 5.8*4.5*5.4 cm heterogeneous lesion at the level of upper vagina suggestive of AV malformation. She underwent angiography which showed massive extravasation from the left internal iliac artery and abnormal vascularity from the right internal iliac artery. The anterior branches of bilateral internal iliac arteries were embolized by gel foam and 300 micron polyvinyalchohol. (Figure1-5) She was symptomatically better but had spotting per vaginum on and off. Her urine pregnancy test was positive before hysterectomy so in suspicion of gestational trophoblastic neoplasia, serum BHCG was advised during workup and it was 1997 mIU/ml and was repeated, and the second value was 2582 mIU/ml. A diagnosis of GTN was confirmed. On further inquiry, she brought one ultrasound report, which showed molar pregnancy before hysterectomy. After that, she had diarrhea, fever, and anemia. One unit packed cell was transfused. She again had an episode of heavy bleeding per vaginum after 1 week. CT angiogram was performed which showed an ill-defined residual cystic lesion measuring 4.8*4.7 cm in the pelvic cavity adjacent to the left iliac vessel. There is dilated vascular channel suggestive of arterio-venous malformation. A repeated embolization of feeders of bilateral internal iliac arteries and selective feeders of right external iliac arteries was done with 300 micrograms of polyvinyl alcohol. She has transfused 4 units of packed red blood cells during her stay.

The patient was further investigated by CECT abdomen and thorax because of GTN. Her WHO prognostic score was 4 for gestational trophoblastic neoplasia. She received two cycles of chemotherapy (Inj. Methotrexate 0.4mg/kg/day for 5 days) 2 weeks apart. Her serum BHCG levels reduced to 83.27 mIU/ml after two cycles of chemotherapy. She was discharged and advised to attend an oncology outpatient department for the third cycle of chemotherapy after 2 weeks. 

## Discussion

Arterio-venous malformation (AVM) most commonly congenital can also be formed after procedures like curettage and hysterectomy or in gestational trophoblastic neoplasia (GTN). The etiology of the AVM, in this case, may be due to the invasive process of the trophoblast and with injury following the operative intervention. Patients with AVM usually present with severe episodes of bleeding and anemia. They fail to respond to medical and conservative treatment. The best way to diagnose these cases will be arteriograms after the exclusion of all other possible etiologies. Ultrasonography with Doppler and MRI can be used for the evaluation of AVM.

GTN is a highly vascular and friable tumor. TAE in these cases can be used for primary treatment and bleeding control as well as before a definitive surgical procedure to decrease blood loss [5]. 

Kyung JinEoh et al. reported a case with active vaginal bleeding managed with TAE. The bleeding was diagnosed to be from vaginal metastasis of choriocarcinoma in the case of molar pregnancy treated with hysterectomy 2 years ago. After undergoing TAE patient was given chemotherapy and had complete remission [6]. In one of the case study, TAE was used in a case of the invasive mole before surgery followed by excision of disease and the uterus was conserved for future fertility [7].

TAE is slightly tricky in GTN cases as compared to postpartum hemorrhage. GTN invades, destroys the blood vessel wall leading to a connection between arteries and veins, subsequently forming AVM [8]. 10-15% of GTN are reported to have AVM, which may even be present after chemotherapy and complete remission [5]. According to Keepanasseril A et al. TAE had an 85.7% success rate in treating GTN and should be used in life-threatening hemorrhage [9]. In cases of large AVM the coil rather than the sponge should be used to embolize the vessel as gelatine sponge may pass through the fistula into the circulation. As done in the present case. There had been concerns about the efficacy of chemotherapy in GTN (which undoubtedly is mainstay treatment) after the tumor feeding vessel is blocked and hence hampering the drug delivery to the tumor. Wang Z et al. proved in their study that chemotherapy after UAE had a good resolution rate [10]. Carlini L et al. even showed decreasing and then normalizing of BHCG after TAE without chemotherapy [11]. 

There are cases of recurrent bleeding even after TAE, which is primarily due to neovascularization, the formation of collaterals, and new AVM [12,13]. As the scenario in the present case a repeated TAE is successful in managing such entity. Even though TAE is not a new technique, its utilization is less [14].

TAE is an effective management and a safer alternative to surgery in life-threatening pelvic hemorrhage in gynecological cases such as post-hysterectomy bleeding or bleeding in GTN or both. The index case also reinforces the importance of BHCG monitoring of all molar pregnancy cases which should be practiced without fail.

## Conclusion

Transcatheter artery embolization is an effective management and a safer alternative to surgery in life-threatening pelvic hemorrhage in gynecological cases such as post-hysterectomy bleeding or bleeding in GTN, or both. It should not only be reserved for elective cases, but if made more technically available in an emergency it is likely to be rewarding for patient care. More than one attempt of TAE might be required to achieve hemostasis in certain atypical cases and an attempt at it shall be contemplated. The index case also reinforces the importance of BHCG monitoring of all molar pregnancy cases which should be practiced without fail.
